# MIMU Optimal Redundant Structure and Signal Fusion Algorithm Based on a Non-Orthogonal MEMS Inertial Sensor Array

**DOI:** 10.3390/mi14040759

**Published:** 2023-03-29

**Authors:** Liang Xue, Bo Yang, Xinguo Wang, Guangbin Cai, Bin Shan, Honglong Chang

**Affiliations:** 1 Department of Control Engineering, Xi’an Research Institute of High Technology, Hongqing Town, No. 2 Tongxin Road, Xi’an 710025, China; 2 Ministry of Education Key Laboratory of Micro and Nano Systems for Aerospace, Northwestern Polytechnical University, No. 127 Youyi West Road, Xi’an 710072, China

**Keywords:** MEMS sensor, redundant MIMU, non-orthogonal array, noise correlation, Kalman filter, performance improvement

## Abstract

A micro-inertial measurement unit (MIMU) is usually used to sense the angular rate and acceleration of the flight carrier. In this study, multiple MEMS gyroscopes were used to form a spatial non-orthogonal array to construct a redundant MIMU system, and an optimal Kalman filter (KF) algorithm was established by a steady-state KF gain to combine array signals to improve the MIMU’s accuracy. The noise correlation was used to optimize the geometric layout of the non-orthogonal array and reveal the mechanisms of influence of correlation and geometric layout on MIMU’s performance improvement. Additionally, two different conical configuration structures of a non-orthogonal array for 4,5,6,8-gyro were designed and analyzed. Finally, a redundant 4-MIMU system was designed to verify the proposed structure and KF algorithm. The results demonstrate that the input signal rate can be accurately estimated and that the gyro’s error can also be effectively reduced through fusion of non-orthogonal array. The results for the 4-MIMU system illustrate that the gyro’s ARW and RRW noise can be decreased by factors of about 3.5 and 2.5, respectively. In particular, the estimated errors (1σ) on the axes of *X_b_*, *Y_b_* and *Z_b_* were 4.9, 4.6 and 2.9 times lower than that of the single gyroscope.

## 1. Introduction

**Figure 1:**
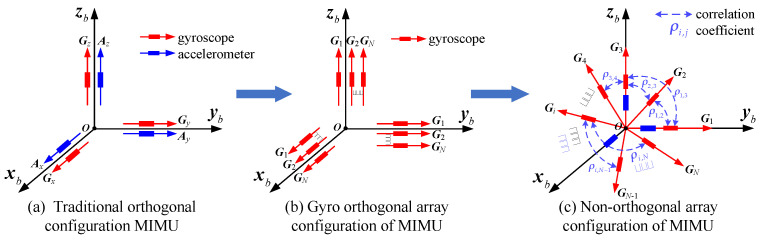
Schematic diagram of the MIMU formed by different MEMS inertial sensors.

The strapdown inertial navigation system has gradually replaced the traditional navigation systems, since the latter are expensive and bulky. Therefore, miniaturization, high accuracy and low cost have become the important features of the modern navigation system. MEMS sensors are suitable for designing a strapdown micro inertial navigation system due to their advantages of a small volume, low cost and high reliability [1,2,3]. The micro-inertial measurement unit (MIMU) is usually composed of three MEMS gyroscopes and accelerometers that are orthogonal to each other (Figure 1a), which is regarded as the core component of an inertial navigation system. It has an important application for miniaturized, high-precision, low-cost navigation and guidance systems. People pay more attention to the continuous improvement of the MIMU while making full use of its unique advantages to meet the urgent needs of high accuracy, miniaturization and low cost for a modern navigation system. However, due to the limitation of the working principle of the MEMS inertial device, the MIMU’s measurement noise is large, and the parameters are also unstable, which makes it difficult to provide inertial signals with low drift error for the MIMU composed of three independent gyroscopes and accelerometers.

The technology of multiple signals fusion provides a new method for reducing the MIMU’s drift error and improving its accuracy [4,5,6]. Compared to an MIMU constructed of three orthogonal sensors and a sensor array (Figure 1a,b), using a non-orthogonal array to design a MIMU can increase its measurement information on the sensors’ coordinate frame, which is mapped from the input rate and acceleration of the MIMU. A fused MIMU can be achieved through fusing redundant measurements while employing a technique of signal fusion and estimation, which can effectively improve the MIMU’s precision. Moreover, the number of sensors can be reduced compared to an MIMU constructed by an orthogonal sensor array, and thus, a performance improvement can be achieved with the same number of chips.

As for the redundant configuration of a MIMUs, Pejsa in 1974 first proposed a method to analyze the construction of a multi-sensor, redundant configuration [7]. In [8], the redundant sensors structures based on different numbers of gyroscopes were studied, and the accuracy of the system’s configuration matrix was analyzed. In addition, a redundant MIMU analysis method was established based on a virtual-body coordinate frame [9]—in particular, a dodecahedron was adopted to construct the redundant structure, which reduced the calculation amount. Jafari used a least-square method to acquire MIMU’s motion information, and then the redundant configurations with three and four gyros were analyzed [10]. In addition, a MIMU system based on an orthogonal gyro array was reported in [11], in which three gyroscope arrays were arranged on the three orthogonal axes of a MIMU, respectively, as shown in Figure 1b; thus, the accuracy was improved through signal fusion. Skog et al. proposed a centralized KF based on maximum likelihood estimation for combining the measurements of a 4 × 4 planar gyroscope array [12]. The redundant gyroscope systems composed of 3, 5 and 8 gyros were designed in [13]. In our previous studies, the configuration structure of redundant systems for 4-, 5- and 6-gyro cones were designed [14]—in particular, the geometric accuracy factor model was established to evaluate structure, and thus the optimal conical installation angle could be obtained. Moreover, the influences of the correlation factor on the geometric accuracy factor and configuration structure were analyzed.

As for the fusion algorithm of a redundant MIMU system, Skog et al. designed multiple signal fusion algorithms for a MIMU planar array using the maximum likelihood estimation method to obtain the optimal estimation of input angular rate and acceleration [15]. A centralized KF was designed for signal fusion of redundant MIMU sensors in [16]. Additionally, an integrated navigation algorithm for combining multiple MIMU and GPS was designed in [17], in which the three-dimensional trajectory dynamics model was used as the system model to design a centralized KF and estimate navigation parameters. In addition, a planar MIMU with 8 × 8 array was formed by 64 chips, and six positions and angular rate position method were used to calibrate the errors and installation angles of gyroscopes and accelerometers in MIMUs [18]; thus, the dynamic navigation performance of a MIMU array can be improved through compensating the output signals of inertial sensors.

The technology of redundant MIMUs have been studied, which combine multiple MIMU arrays to improve accuracy; however, little research has focused on designing a redundant MIMU using a non-orthogonal sensor array. In particular, the correlation of the sensor array has not been incorporated to optimize its geometric structure, which lacks the theoretical support for optimal configuration design under the condition of noise correlation. Compared with a MIMU system composed of multiple MIMUs, using a non-orthogonal sensor array to construct a redundant MIMU provides greater flexibility in spatial layout. In addition, the correlation will affect the optimal configuration structure of the non-orthogonal array and its accuracy. Consequently, it is important to optimize the configuration structure by incorporating the sensor’s noise correlation.

Therefore, in this study, multiple MEMS gyroscopes were used to form a spatial non-orthogonal array to construct a redundant MIMU (Figure 1c). In particular, the noise correlation was used to optimize the geometric layout of a non-orthogonal sensor array, and it revealed the mechanisms of influence of correlation and geometric layout on the MIMU’s performance improvement. The work includes three parts: (1) A KF model is presented for fusing the array’s redundant measurements, and then a KF algorithm is also established to estimate the optimal signal rate in the MIMU’s body coordinate frame. (2) Different configuration structures of a non-orthogonal array for the MIMU are designed, and the influences of the number of sensors (*N*), installation angle (*α*) and correlation factors (*ρ*) on the conical configuration structures are analyzed. (3) Simulations and experiments are reported to verify the presented algorithm.

## 2. Working Principle of a Redundant MIMU System

**Figure 2:**
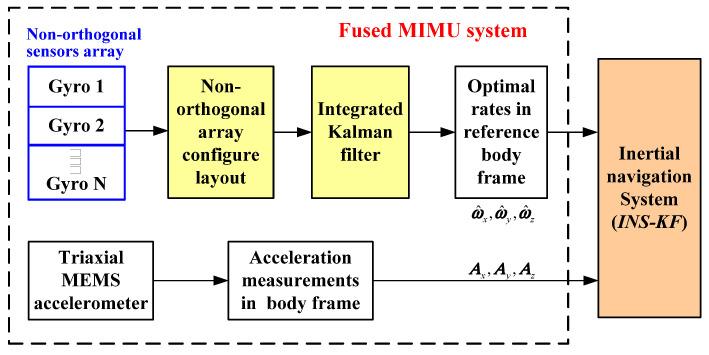
Structure of a redundant fused MIMU system.

The principle of a redundant MIMU is shown in Figure 2. Multiple gyroscopes are used to form a non-orthogonal array in three-dimensional space, and three orthogonal accelerometers are arranged in the MIMU’s body coordinate frame. Then, an optimal KF is designed to fuse outputs of non-orthogonal gyroscope array, and thus the optimal estimation of the input rate in the MIMU’s body coordinate frame can be obtained by using the redundant structure and its configuration matrix. The redundant fused MIMU system can be completed with three orthogonal accelerometers.

### 2.1. Modeling of a Fused-Gyro-Array KF Algorithm

A typical measurement model is used to describe the gyroscope [5,6]:

(1) $$\left\{ \begin{array}{l} y(t)=\omega (t)+b(t)+n(t)\\ \dot{b}(t)=w_{b}(t) \end{array}\right.$$

where *y* is the output of the gyroscope, *ω* is the input signal rate denoted as the true rate, *b* is the gyro’s drift due to the rate random walk (RRW) process *w_b_* and *n* is the angular random walk’s (ARW) white noise.

The KF was employed to design a fused algorithm for the non-orthogonal array. Given a gyroscope array with the number of sensors *N*, according to Equation (1), the measurement model of the non-orthogonal array is:

(2) $$y=H\omega^{b}+b+n$$

where **H** is the configuration matrix of the non-orthogonal gyroscope array, which can be determined by the specific structure of the sensor array, $y={\left[y_{1},y_{2},\cdots ,y_{N}\right]}^{T}$ is the measurement vector of non-orthogonal gyroscope array, $\omega^{b}={\left[\omega_{x}^{b},\omega_{y}^{b},\omega_{z}^{b}\right]}^{T}$ is the input rate in the MIMU’s body frame, $b={\left[b_{1},b_{2},\cdots ,b_{N}\right]}^{T}$ is the gyro’s drift error and $n={\left[n_{1},n_{2},\cdots ,n_{N}\right]}^{T}$ is the white noise. The input true signal rate can be modeled directly; thus, its optimal estimate can be directly obtained using the KF. In addition, the accuracy of the fused signal rate can be analyzed by the KF covariance, which also can provide a basis for system improvement and parameter adjustment. Therefore, to build a complete state-space model for the KF and improve accuracy, the input signal rate **ω***^b^* can be modeled using a random walk process driven by white noise **n***_ωr_* [4,6]:

(3) $${\dot{\omega }}^{b}=n_{\omega r}$$

where the driven white noise vector $n_{\omega r}={\left[n_{\omega rx},n_{\omega ry},n_{\omega rz}\right]}^{T}$, and $E[n_{\omega r}(t)]=0$, $E[n_{\omega r}(t)n_{\omega r}^{T}(t+\tau )]=q\delta (\tau )$. The component values of matrix **q** should be determined by the gyro’s noise level and the dynamic characteristic requirement of the input signal rate. From a practical point of view, the application could be satisfied by choosing an appropriate variance *q_ωx_*_,*y*,*z*_ with which to control the different bandwidths of the KF. Here, the angular rate **ω***^b^* and drift vector **b** were chosen to construct the KF state vector as $X=[\omega^{b};\;b]$; the measurement was selected as **Z** = **y**. Based on Equations (2) and (3), the state-space model of the non-orthogonal gyroscope array can be formed as:

(4) $$\left\{ \begin{array}{l} \dot{X}(t)=F(t)X(t)+W(t)\\ Z(t)=H_{1}X(t)+V(t) \end{array}\right.$$

where coefficient matrix $F=0_{\left(N+3)\times (N+3\right)}$, $H_{1}=\left[H\vdots I_{N}\right]$, the system’s process noise $W(t)={\left[n_{\omega r},w_{b}\right]}^{T}$ and measurement noise $V(t)={\left[n_{1},n_{2},\cdots ,n_{N}\right]}^{T}$. The covariance matrix of the vectors **W**(*t*) and **V**(*t*) are **Q** and **R**, respectively. The matrices **Q** and **R** are not necessarily diagonal because of the gyro’s noise correlation; thus, the matrices **Q**, **R** and **q** are given in Equation (5).

(5) $$Q=\left[\begin{array}{cc} q & 0_{3\times N}\\ 0_{N\times 3} & Q_{b} \end{array}\right],\;q=\left[\begin{array}{ccc} q_{\omega x} & 0 & 0\\ 0 & q_{\omega y} & 0\\ 0 & 0 & q_{\omega z} \end{array}\right]\;R={\left[\begin{array}{cccc} \sigma_{n1}^{2} & \rho_{n,}{}_{12}\cdot \sqrt{\sigma_{n1}^{2}\sigma_{n2}^{2}} & \cdots & \rho_{n}{}_{,1N}\cdot \sqrt{\sigma_{n1}^{2}\sigma_{nN}^{2}}\\ \rho_{n,}{}_{21}\cdot \sqrt{\sigma_{n2}^{2}\sigma_{n1}^{2}} & \sigma_{n2}^{2} & \cdots & \rho_{n,}{}_{2N}\cdot \sqrt{\sigma_{n2}^{2}\sigma_{nN}^{2}}\\ \vdots & \vdots & \ddots & \vdots \\ \rho_{n}{}_{,N1}\cdot \sqrt{\sigma_{nN}^{2}\sigma_{n1}^{2}} & \rho_{n,}{}_{N2}\cdot \sqrt{\sigma_{nN}^{2}\sigma_{n2}^{2}} & \cdots & \sigma_{nN}^{2} \end{array}\right]}_{N\times N}\;Q_{b}={\left[\begin{array}{cccc} \sigma_{b1}^{2} & \rho_{b,12}\cdot \sqrt{\sigma_{b1}^{2}\sigma_{b2}^{2}} & \cdots & \rho_{b,1N}\cdot \sqrt{\sigma_{b1}^{2}\sigma_{bN}^{2}}\\ \rho_{b,21}\cdot \sqrt{\sigma_{b2}^{2}\sigma_{b1}^{2}} & \sigma_{b2}^{2} & \cdots & \rho_{b,2N}\cdot \sqrt{\sigma_{b2}^{2}\sigma_{bN}^{2}}\\ \vdots & \vdots & \ddots & \vdots \\ \rho_{b,N1}\cdot \sqrt{\sigma_{bN}^{2}\sigma_{b1}^{2}} & \rho_{b,N2}\cdot \sqrt{\sigma_{bN}^{2}\sigma_{b2}^{2}} & \cdots & \sigma_{bN}^{2} \end{array}\right]}_{N\times N}$$

where $\sigma_{bi}^{2}$ and $\sigma_{ni}^{2}$ are the noise variances of RRW and ARW associated with the ith gyro in the array, respectively; and *ρ_ij_* is the correlation factor between the ith and jth gyros, and the practical value of correlation factor *ρ_ij_* can be analyzed and obtained by the method referred in [19]. The parameters *q_ωx_*, *q_ωx_* and *q_ωz_* are the variances of white noise **n***_ωr_*, which drive the input rate **ω***^b^*. Based on Equation (4), the continuous-time KF algorithm for the non-orthogonal gyroscope array is

(6) $$\left\{ \begin{array}{l} \dot{\hat{X}}(t)=K(t)\left[Z(t)-H_{1}\hat{X}(t)\right]\\ K(t)=P(t)H_{1}^{T}R^{-1}\\ \dot{P}(t)=Q-P(t)H_{1}^{T}R^{-1}H_{1}P(t) \end{array}\right.$$

**Figure 3:**
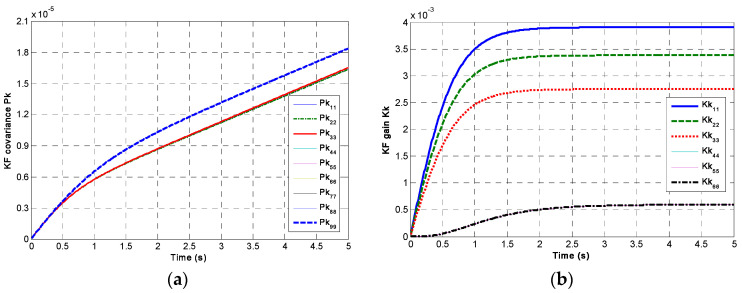
The plot of KF covariance **P**(*t*) and gain **K**(*t*): (**a**) **P**(*t*); (**b**) **K**(*t*).

The rank of the KF’s observability matrix is *N*, which is lower than its dimensions of *N* + 3; thus, the KF system (**F**,**H**_1_) is not completely observable, and there is no steady-state solution to **P**(*t*) in Equation (6). Here, set the gyro’s noise variance to be *σ_b_* = 600°/h/$\sqrt{h}$ and *σ_n_* = 2°/$\sqrt{h}$, then choose signal sampling period *T* = 0.01 s and correlation factor *ρ* = 0. For the non-orthogonal array composed of six gyros, the changes in covariance **P**(*t*) and gain **K**(*t*) are shown in Figure 3.

It can be seen in Figure 3 that the component of matrix **P***_k_* will be linearly increased and divergent with the iteration time. It does not have a steady-state solution. However, the matrix **K***_k_* tends toward a steady-state value in a short time, which indicates a steady-state gain **K***_s_* can be obtained. Using the steady gain **K***_s_* can simplify the implementation of the KF system. It does not need to calculate covariance **P**(*t*) in each iteration, which reduces the computational load. Therefore, for a redundant MIMU system with a determined structure of non-orthogonal array, a steady-state gain **K***_s_* can be obtained offline by the discrete equation of KF. Thus, Equation (6) can be written as:

(7) $$\dot{\hat{X}}(t)=K_{s}(t)\left[Z(t)-H_{1}\hat{X}(t)\right]$$

By discretizing Equation (7), the KF discrete equation can be obtained:

(8) $${\hat{X}}_{k+1}=e^{-K_{s}H_{1}T}{\hat{X}}_{k}+\int_{0}^{T}e^{-K_{s}H_{1}t}dtK_{s}Z_{k}$$

Define matrix **K***_H_* = **K***_s_***H**_1_, and perform an eigenvalue decomposition for the matrix **K***_H_* as **K***_H_*= **SΛS**^−1^, where the columns of matrix **S** are composed of the eigenvectors of matrix **K***_H_*. **Λ** is a diagonal matrix composed of the eigenvalues of **K***_H_*, $e^{-K_{H}T}=e^{-S\Lambda S^{-1}T}=Se^{-\Lambda T}S^{-1}$ and $\int_{0}^{T}e^{-K_{H}T}dt=\int_{0}^{T}e^{-S\Lambda S^{-1}T}dt=S\int_{0}^{T}e^{-\Lambda T}dtS^{-1}$; thus, the discrete Equation (8) can be formed as:

(9) $${\hat{X}}_{k+1}=Se^{-\Lambda T}S^{-1}\cdot {\hat{X}}_{k}+S\int_{0}^{T}e^{-\Lambda T}dtS^{-1}\cdot K_{s}Z_{k}$$

**Figure 4:**
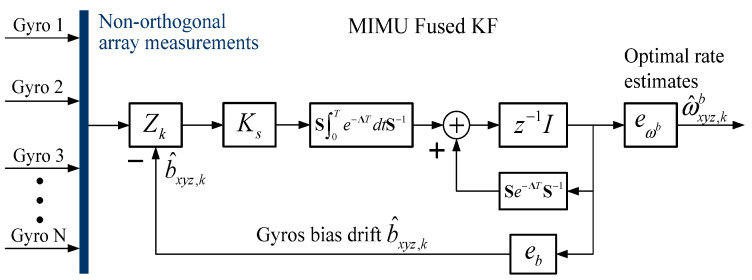
Redundant MIMU signal fusion using a discrete-time KF implementation.

By defining the rate extract vector $e_{\omega b}=\left[I_{3}\vdots O_{3\times N}\right]$, the optimal estimation of rate in MIMU’s body coordinate frame can be obtained as ${\hat{\omega }}_{xyz,k+1}^{b}=e_{\omega b}\cdot {\hat{X}}_{k+1}$. The discrete KF structure is shown in Figure 4. Consequently, the optimal estimation of orthogonal rate for the MIMU system can be obtained by using the discrete iterative estimation equation, Equation (9).

### 2.2. Structure of a Non-Orthogonal Gyro Array in MIMU

**Figure 5:**
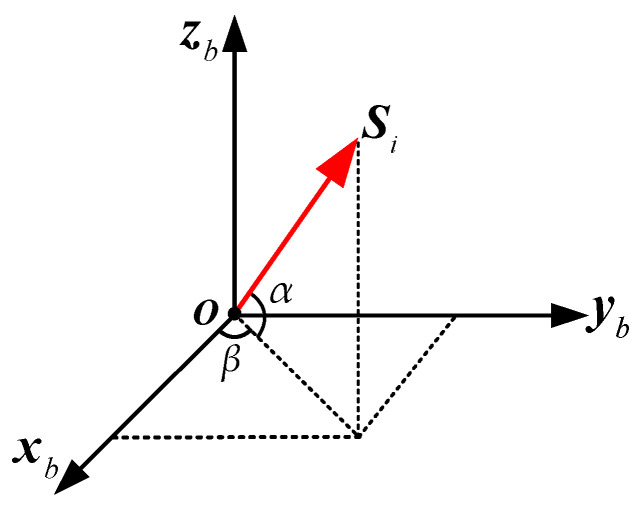
Installation schematic diagram of the component gyroscopes.

In the fused MIMU system, the installation of individual gyroscopes in the non-orthogonal array is shown in Figure 5, where *S_i_* is the unit vector of the ith gyro’s sensitive axis. It can be formed as:

(10) $$S_{i}=\cos\alpha_{i}\cdot \cos\beta_{i}i+\cos\alpha_{i}\cdot \sin\beta_{i}j+\sin\alpha_{i}k$$

where *α_i_* and *β_i_* are the installation angles of the ith gyro relative to the MIMU’s body coordinate frame (*X_b_*, *Y_b_*, *Z_b_*). According to Equation (10), the configuration matrix of a non-orthogonal array can be expressed as:

(11) $$H=\left[\begin{array}{ccc} \cos\alpha_{1}\cdot \cos\beta_{1} & \cos\alpha_{1}\cdot \sin\beta_{1} & \sin\alpha_{1}\\ \cos\alpha_{2}\cdot \cos\beta_{2} & \cos\alpha_{2}\cdot \sin\beta_{2} & \sin\alpha_{2}\\ \vdots & \vdots & \vdots \\ \cos\alpha_{N}\cdot \cos\beta_{N} & \cos\alpha_{N}\cdot \sin\beta_{N} & \sin\alpha_{N} \end{array}\right]$$

**Figure 6:**
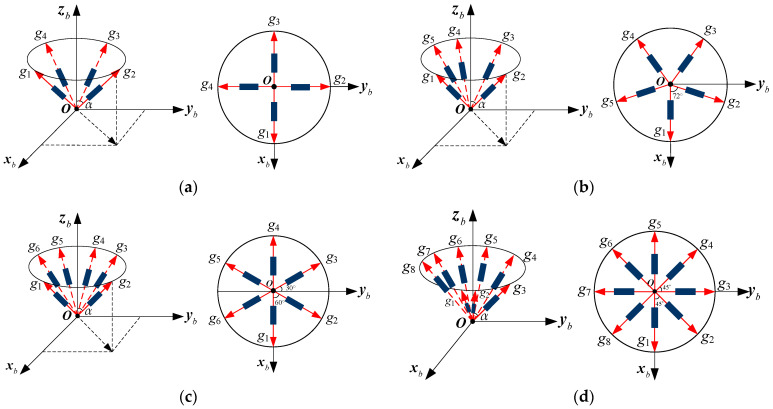
Non-orthogonal gyro array for a MIMU without a one-axis conical sensor: (**a**) 4-gyro cone; (**b**) 5-gyro cone; (**c**) 6-gyro cone; (**d**) 8-gyro cone.

For different numbers of sensors, *N*, the MIMU configurations differ, corresponding to different matrices, **H**. The conical configuration is a typical structure for a non-orthogonal array. In this paper, two configuration schemes for a conical structure are designed and analyzed with *N* = 4, 5, 6, 8, which is illustrated as follows:

Scheme 1: Multiple gyroscopes are arranged as cone and evenly distributed around the MIMU’s body frame along the *Z_b_* axis (in Figure 6). Specifically, each gyroscope is evenly installed and distributed on the cone’s surface, and its sensitive axis is along an imaginary line connecting the tip of the cone and the gyro. The angle between each gyro’s sensitive axis and the +*Z_b_* axis is *α*. Figure 6a is a structure with *N* = 4, in which the angles between the +*X_b_* axis and projections of g1, g2, g3 and g4’s sensitive axes on the horizontal plane are 0°, 90°, 180° and 270°, respectively. For *N* = 5, the projection of g1’s sensitive axis on the horizontal plane coincides with the +*X_b_* axis, and the angle between the projections of contiguous gyros’ sensitive axes is 72°, which is shown in Figure 6b. In addition, for *N* = 6 in Figure 6c, the projections of g1 and g4’s sensitive axes on the horizontal plane coincide with +*X_b_* and −*X_b_*, respectively—in particular, the angle between the projections of contiguous gyros’ sensitive axes on the horizontal plane is 60°. For *N* = 8 in Figure 6d, the angle between the projections of contiguous gyros’ sensitive axes on the horizontal plane is 45°.

**Figure 7:**
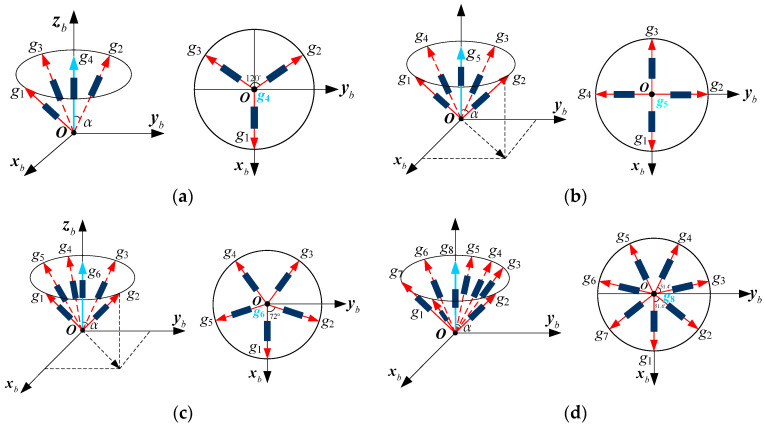
Non-orthogonal gyro array for the MIMU with a one-axis conical sensor: (**a**) 4-gyro cone; (**b**) 5-gyro cone; (**c**) 6-gyro cone; (**d**) 8-gyro cone.

Scheme 2: Multiple gyroscopes are arranged as a cone and evenly distributed around the MIMU’s body frame’s *Z_b_* axis—in particular, one gyro’s sensitive axis coincides with the axis +*Z_b_*, and the other gyroscopes are evenly distributed around the +*Z_b_* axis, as shown in Figure 7. Specifically, the angle between the gyro’s sensitive axis and +*Z_b_* axis is *α*, and the projection of g1’s sensitive axis on the horizontal plane coincides with axis +*X_b_*. For *N* = 4 in Figure 7a, the angles between the +*X_b_* axis and projections of g1, g2 and g3’s sensitive axes on the horizontal plane are 0°, 120°, 240°, respectively. For *N* = 5, 6 and 8, the angles between the projections of contiguous gyros’ sensitive axis on the horizontal plane are 90°, 72° and 360/7°, respectively, which are shown in Figure 7b–d.

For the different conical configurations, the estimated accuracy of signal fusion is different, even for identical values of *N*. The geometric accuracy factor (GDOP) is usually used to evaluate the quality of a redundant configuration structure in Equation (12) [14]. The smaller the GDOP, the better the redundant configuration structure, and thus the estimated accuracy of signal fusion will be higher:

(12) $$GDOP=\sqrt{tr{\left(H_{1}^{T}C_{n}^{-1}H_{1}\right)}^{-1}},\;C_{n}={\left[\begin{array}{cccc} 1 & \rho_{n,}{}_{12} & \cdots & \rho_{n}{}_{,1N}\\ \rho_{n,}{}_{21} & 1 & \cdots & \rho_{n,}{}_{2N}\\ \vdots & \vdots & \ddots & \vdots \\ \rho_{n}{}_{,N1} & \rho_{n,}{}_{N2} & \cdots & 1 \end{array}\right]}_{N\times N}$$

where **H**_1_ is the measurement matrix for KF in MIMU and **C***_n_* is the cross-correlation matrix associated with the ARW noise of the non-orthogonal gyroscope array.

## 3. Performance of Configuration Structure of Non-Orthogonal Array

The performance of the MIMU’s configuration structure is affected by many factors. From Equation (12), it can be seen that it is affected by the number *N*, configuration structure (i.e., sensor’s installation angle *α*) and correlation factor. Therefore, as for the two schemes of redundant configurations shown in Figure 6 and Figure 7, the factors affecting the optimal redundant configuration were analyzed through the GDOP under the condition of noise correlation in the sensor array. Eventually, the optimal installation angle *α* and correlation factor *ρ* could be determined. Firstly, the influence of *N* on the GDOP is analyzed. Given the correlation factor in the gyroscope array is 0, when the installation angle, *α*, is set to 60° or 45°, the GDOP results for the different configuration structures in Figure 6 and Figure 7 are shown in Table 1.

**Figure 8:**
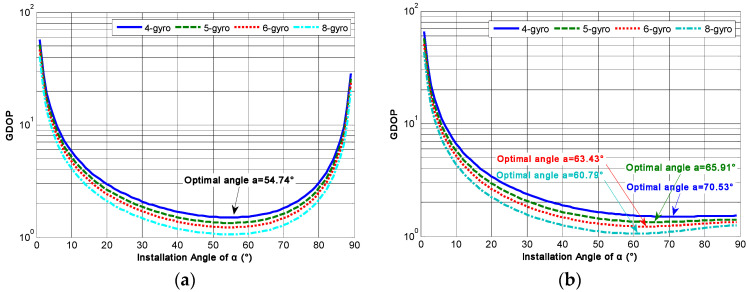
The relationship between GDOP and angle *α* for different structures (*ρ* = 0). (**a**) Configuration Scheme 1; (**b**) configuration Scheme 2.

Table 1 illustrates that the GDOP will decrease with increasing *N* under the identical configuration structure, which indicates that the estimated accuracy of the three-dimensional orthogonal signal rate in the MIMU’s body coordinate frame will be improved with increasing *N*. Furthermore, in order to obtain the optimal *α* for the conical non-orthogonal array, the relationship between the GDOP and angle *α* for different conical configuration structures are analyzed by Equation (12). It can be seen that the correlation factor will influence the optimal angle *α*. When the correlation factor *ρ* = 0, the relationship between the GDOP and angle *α* for a non-orthogonal array with different values of *N* is illustrated in Figure 8.

As for configuration Scheme 1, from Figure 8a it can be found that the GDOP will decrease as *α* increases. After reaching a minimum value, it will then gradually increase, and the angle *α* corresponding to such a minimum value is the optimal installation angle for the conical structure. The minimum GDOPs are 1.5001, 1.3417, 1.2248 and 1.0607 for *N* = 4, 5, 6 and 8 respectively, and the optimal *α* is 54.74°, which indicates that the identical configuration structures have the same optimal installation angle; *ρ* = 0 even if *N* is different. On the other hand, for configuration Scheme 2, Figure 8b illustrates that the GDOP will gradually decrease as *α* increases and then slowly increase. Eventually, it will gently decline until it reaches a minimum value. The results show that the minimum GDOPs were 1.5000, 1.3416, 1.2247 and 1.0607 for *N* = 4, 5, 6 and 8, respectively; and the corresponding optimal angles were 70.53°, 65.91°, 63.43° and 60.79° respectively. This indicates that the optimal angles α will decrease with increasing *N* for configuration Scheme 2.

**Figure 9:**
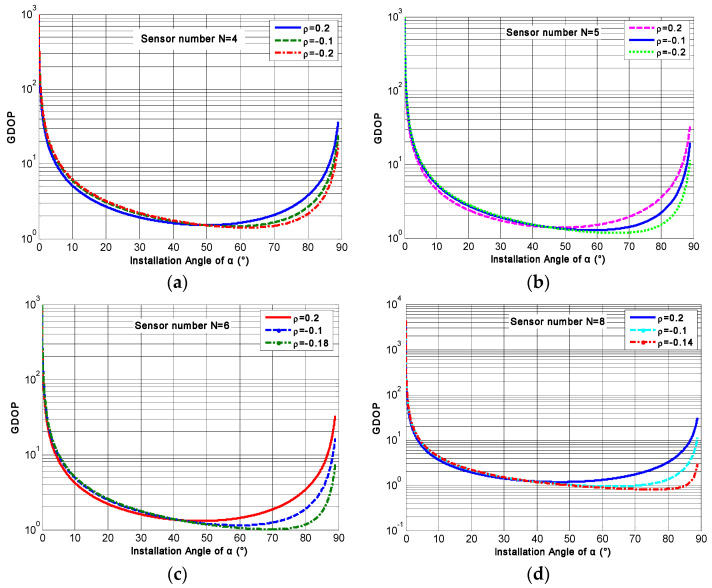
The relationship between GDOP and angle *α* for different *N* with various correlation factors: (**a**) *N* = 4; (**b**) *N* = 5; (**c**) *N* = 6; (**d**) *N* = 8.

As for conical configuration Scheme 1, the relationship between the GDOP and installation angle *α* with different correlation factors was further analyzed. The results are illustrated in Figure 9, and then the optimal angle *α* under such a correlation factor can be obtained from the plot. The results of optimal angle *α* are listed in Table 2. It can be seen that the optimal angle *α* corresponding to the minimum GDOP will be different with different correlation factors. In addition, Table 2 also shows that the optimal angle *α* is different for different *N*, even if the correlation factor is the same.

**Figure 10:**
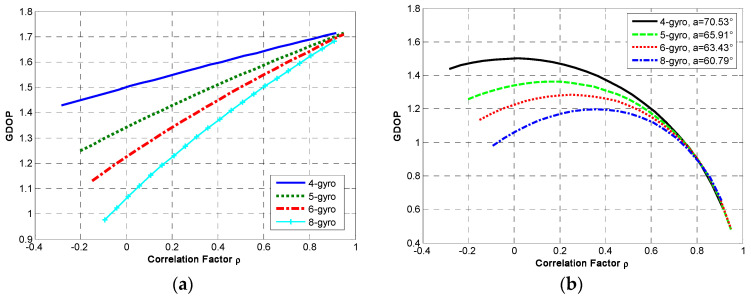
The relationship between correlation factor and GDOP. (**a**) Configuration Scheme 1; (**b**) configuration Scheme 2.

Additionally, Equation (12) shows that the accuracy of redundant MIMU is also affected by the noise correlation in the sensor array. It is assumed that a constant correlation factor *ρ_n_* exists in the non-orthogonal array, and its range is [−1/(*N* − 1),1]. The relationship between the correlation factor *ρ_n_* and GDOP is analyzed, and the result is shown in Figure 10.

The following can be seen in Figure 10a: (1) The GDOP is positively correlated with *ρ_n_* and will increase as factor *ρ_n_* increases. (2) The effect of *ρ_n_* on the GDOP depends on *N*. Concretely, the GDOP will shrink as *N* increases under the same factor *ρ_n_*, leading to a higher performance. This also verifies that the system’s accuracy will be higher with a higher *N* for an identical configuration structure. (3) The effect of *ρ_n_* on GDOP is different. The KF’s accuracy with a negative *ρ_n_* is higher than that with a positive one, which indicates that the smaller the correlation factor *ρ_n_*, the better the configuration structure. On the other hand, Figure 10b shows that: (1) The GDOP will increase as factor *ρ_n_* increases, reaches a maximum value and then gradually decreases. (2) The maximum value of GDOP will decrease as *N* increases with the optimal angle α. In addition, Figure 10 indicates that the influence of *ρ_n_* on GDOP is equivalent for the same configuration structure, and its influence is different for various configuration structures.

It should be noted that an orthogonal MIMU is usually composed of three gyroscopes and accelerometers that are orthogonal to each other. According to the GDOP formula of Equation (12), it is found that the GDOP is equal to $\sqrt{3}$ ≈ 1.7321 no matter what the correlation factor *ρ_n_* is, which indicates that the GDOP for the orthogonal MIMU is independent of the correlation factor, and the correlation factor has no effect on the orthogonal MIMU’s configuration.

## 4. Simulation Results and Discussion

### 4.1. Results of the Static Simulation

**Figure 11:**
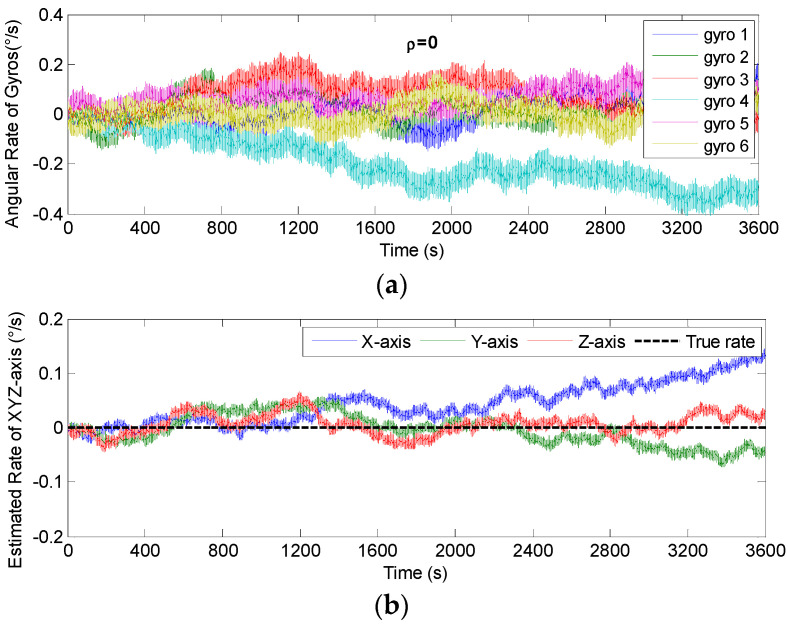
Fused results of the 6-gyro MIMU system for Scheme 1 (*ρ* = 0). (**a**) The output of gyros. (**b**) The estimated result of the MIMU.

**Figure 12:**
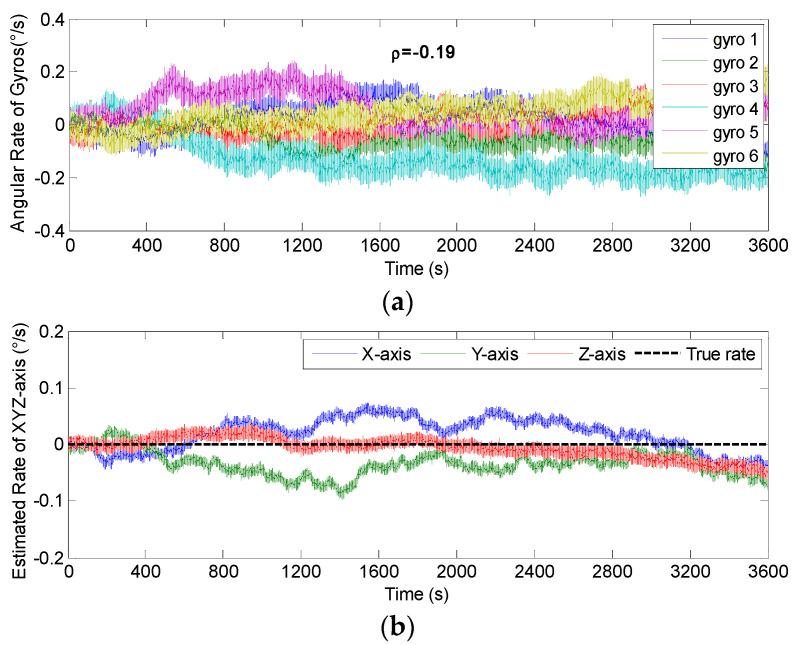
Fused results of the 6-gyro MIMU system for Scheme 1 (*ρ* = −0.19). (**a**) The output of gyros. (**b**) The estimated result of the MIMU.

**Figure 13:**
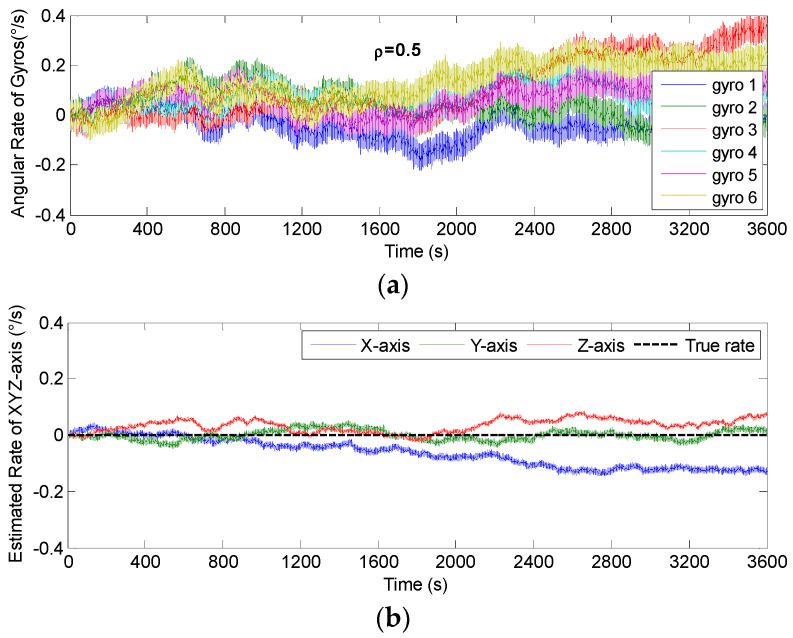
Fused results of 6-gyro MIMU system for Scheme 1 (*ρ* = 0.5). (**a**) The output of gyros. (**b**) The estimated result of the MIMU.

**Figure 14:**
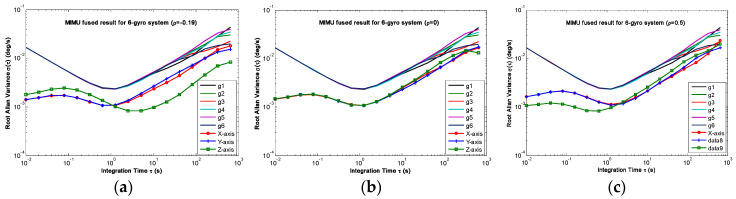
Plot of the compared Allan variance of 6-gyro MIMU for Scheme 1: (**a**) *ρ* = −0.19; (**b**) *ρ* = 0; (**c**) *ρ* = 0.5.

**Figure 15:**
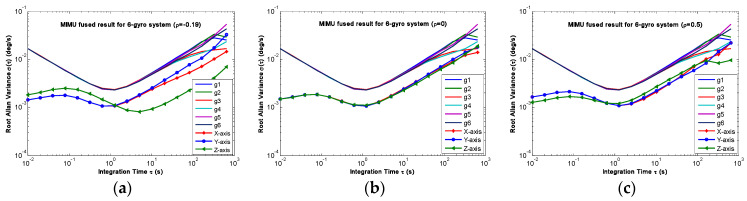
Plot of the compared Allan variance of 6-gyro MIMU for Scheme 2: (**a**) *ρ* = −0.19; (**b**) *ρ* = 0; (**c**) *ρ* = 0.5.

The non-orthogonal array of *N* = 6 in Figure 6c and Figure 7c was chosen to analyze the KF’s performance. The gyro’s ARW and RRW were set as *σ_n_* = 0.1°*/*$\sqrt{h}$ and *σ_b_* = 600°/h/$\sqrt{h}$, respectively. The simulation time and signal sampling period were set to *T* = 1 h and *T_s_* = 0.01 s. The correlation factor for RRW noise was chosen as *ρ_b_* = {−0.19, 0, 0.5}. As for the conical configuration structure in Figure 6c, the estimated rate in MIMU’s body frame is shown in Figure 11, Figure 12 and Figure 13. The Allan variance is shown in Figure 14. The result is listed in Table 3. Additionally, for the structure with *N* = 6 in Figure 7c, the Allan variance is shown in Figure 15, and the result is given in Table 4.

For the conical configuration structure in Scheme 1, it can be seen in Figure 11, Figure 12 and Figure 13 that the rate in MIMU’s body frame can be estimated well, and the gyro’s error can also be reduced. Moreover, Figure 14 shows that the Allan variance curve for estimated rate is lower than that of the single gyroscope, which indicates a remarkable noise reduction. In addition, Table 3 shows that the noise coefficients of estimated rate differ with the various values of factor *ρ*—specifically, the ARW and RRW obtained at *ρ* = 0.19 are lower than those for *ρ* = {0, 0.5}. Thus, the estimation accuracy will be improved as *ρ* decreases, which is accordance with the results in Figure 10a, and the gyro’s ARW and RRW are reduced by a factor about 2.5. On the other hand, as for the conical configuration structure of Scheme 2, Figure 15 and Table 4 demonstrate that the accuracy is also improved through the signals’ fusion. Compared with the results in Table 3 and Table 4, it can be seen that the ARW and RRW of estimated rate for Schemes 1 and 2 are comparable, while *ρ* = 0. This is because the GDOPs of Schemes 1 and 2 are approximately equal while *ρ* = 0 in Figure 10.

### 4.2. Results of the Sinusoidal Signal Simulation

**Figure 16:**
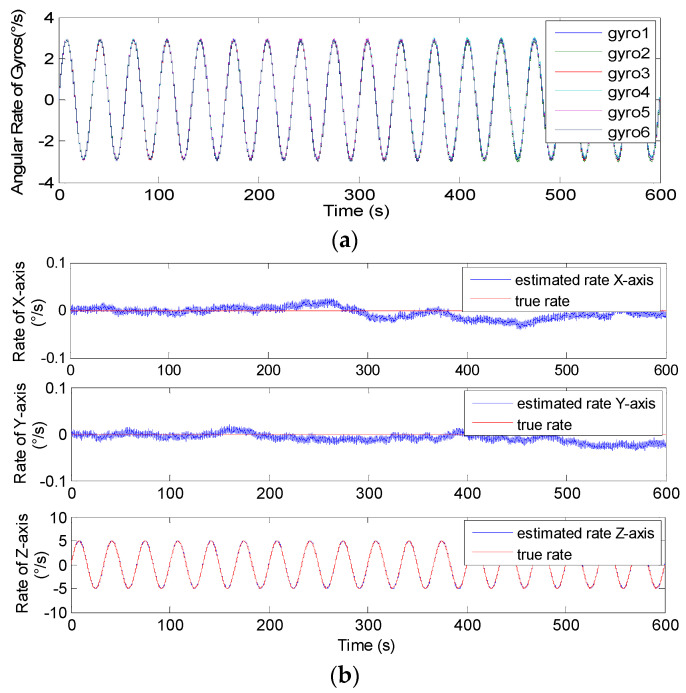
Sinusoidal results of the 6-gyro MIMU system for Scheme 1. (**a**) The outputs of the gyros. (**b**) The estimated results of the MIMU.

**Figure 17:**
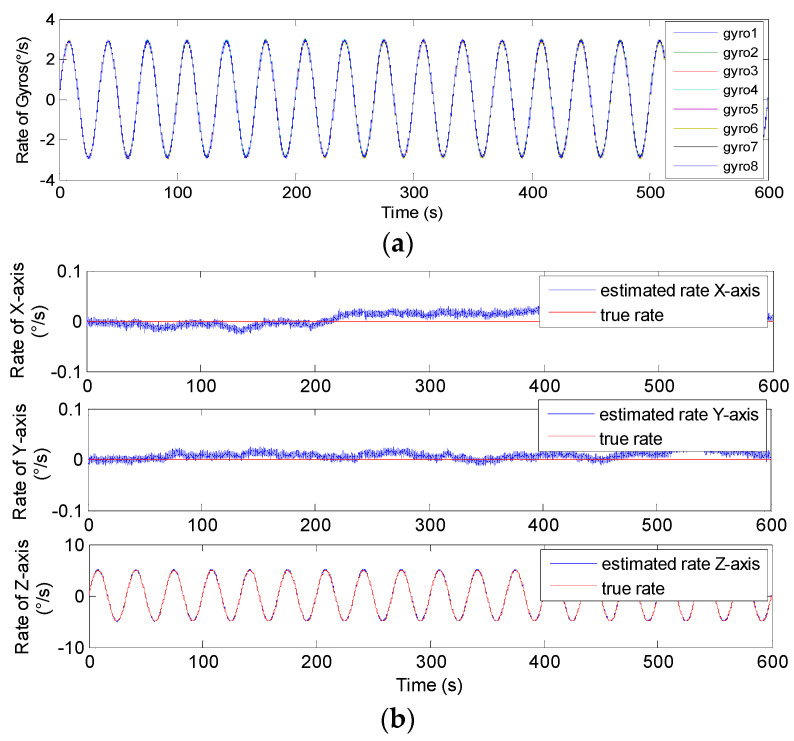
Sinusoidal results of the 8-gyro MIMU system for Scheme 1. (**a**) The outputs of the gyros. (**b**) The estimated results of the MIMU.

**Figure 18:**
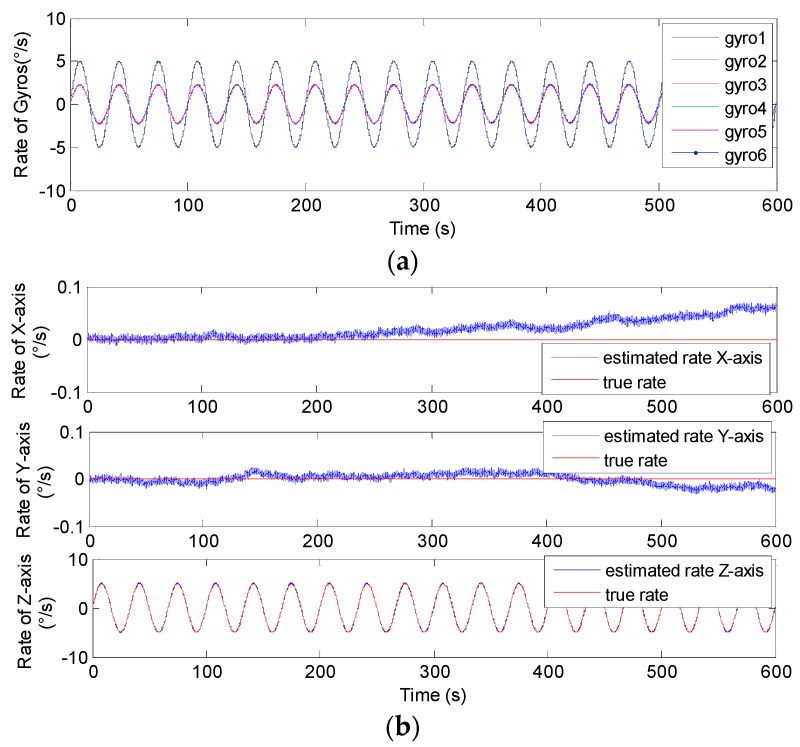
Sinusoidal results of the 6-gyro MIMU system for Scheme 2. (**a**) The outputs of the gyros. (**b**) The estimated results of the MIMU.

**Figure 19:**
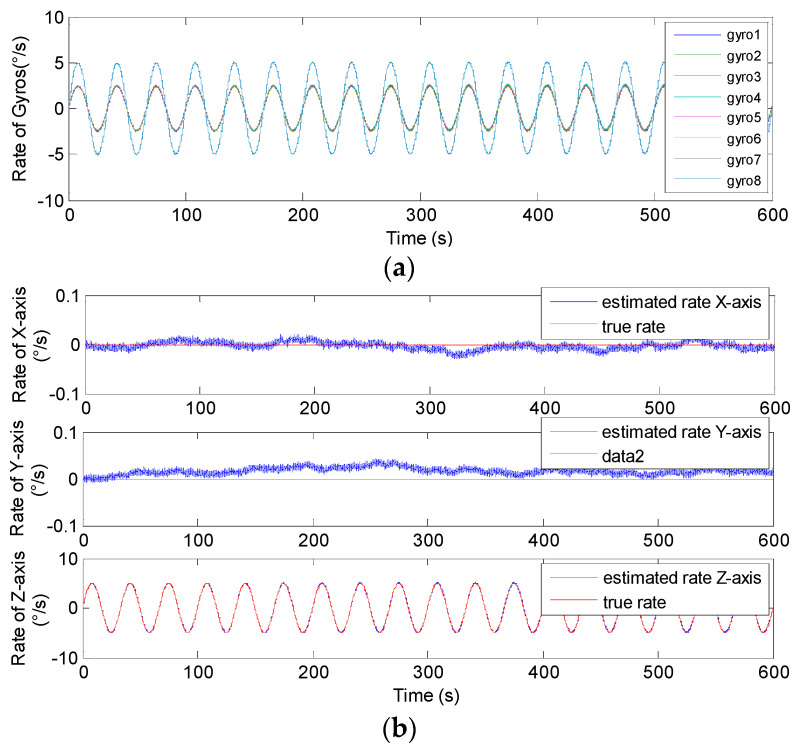
Sinusoidal results of the 8-gyro MIMU system for Scheme 2. (**a**) The outputs of the gyros. (**b**) The estimated results of the MIMU.

The conical configuration structures of *N* = 6,8 in Figure 6 and Figure 7 were chosen to implement the sinusoidal simulation. The input sinusoidal rate was set to be **ω***^b^*= [0, 0, 5 × sin(0.06π*t*)]*^T^*°/s. The gyro’s ARW and RRW were set as *σ_n_* = 0.1°/$\sqrt{h}$ and *σ_b_* = 600°/h/$\sqrt{h}$, respectively, and the correlation factor was set to *ρ* = 0. The simulation time and signal sampling period were set to *T* = 1/6 h and *T_s_* = 0.01 s. For the conical structures of *N* = 6, 8 in Figure 6c,d, the gyro’s installation angle *α* was chosen as 54.74°, and *α* was chosen as 63.43° or 60.79° for *N* = 6,8 in Figure 7c,d, for Scheme 2, respectively. The plots of the non-orthogonal array and estimated signal rate are shown in Figure 16, Figure 17, Figure 18 and Figure 19. The results are listed in Table 5 and Table 6.

In Figure 16, Figure 17, Figure 18 and Figure 19, it can be seen that the amplitude of estimated signal rate on the *Z_b_* axis reaches the input signal of 5°/s without attenuation and distortion. Furthermore, Table 5 and Table 6 show the 1*σ* on the *X_b_* and *Y_b_* axes are about 3 to 9 times lower than those of a single gyroscope, and about 3 times lower on the *Z_b_* axis, which indicates that the accuracy is significantly improved through signal fusion of the non-orthogonal array. Particularly, it clearly shows that the reduction factor of the estimated error for *N* = 8 is higher than that for *N* = 6 for the conical configuration in Schemes 1 and 2, respectively. This also explains that for the same installation angle *α* and correlation factor *ρ*, the larger the value of *N*, the smaller the value of GDOP, and the higher the system fusion and estimation accuracy that can be achieved.

## 5. Experiment

**Figure 20:**
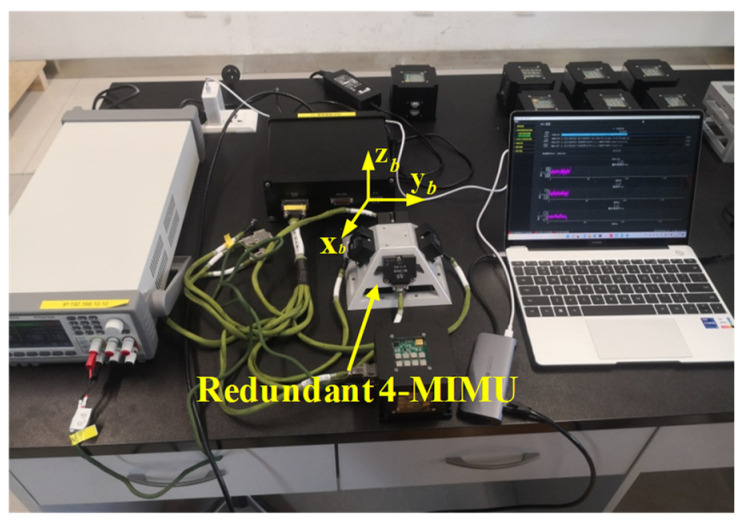
The conical configuration of a 4-MIMU system on the tetrahedral pyramid.

Four individual identical MIMUs were selected to design a redundant MIMU system (Figure 20), which were installed on the four sides of a tetrahedral pyramid. For the individual MIMUs, the *x* axis was defined to be located on the perpendicular bisector of the bottom line of the tetrahedron pyramid, and the downward direction was the axis of +*X*. Here, four *x*-axis gyros from each single MIMU were chosen to form a non-orthogonal array. Using the method in [19], the cross-correlation matrix of a redundant 4-MIMU can be obtained and shown in Table 7, which indicates that the values of correlation factors are close to zero; thus, such a redundant MIMU can be considered uncorrelated. Consequently, the angle between tetrahedron’s each side and undersurface was set as 54.74° according to the results of Figure 8a.

### 5.1. Static Testing Results

**Figure 21:**
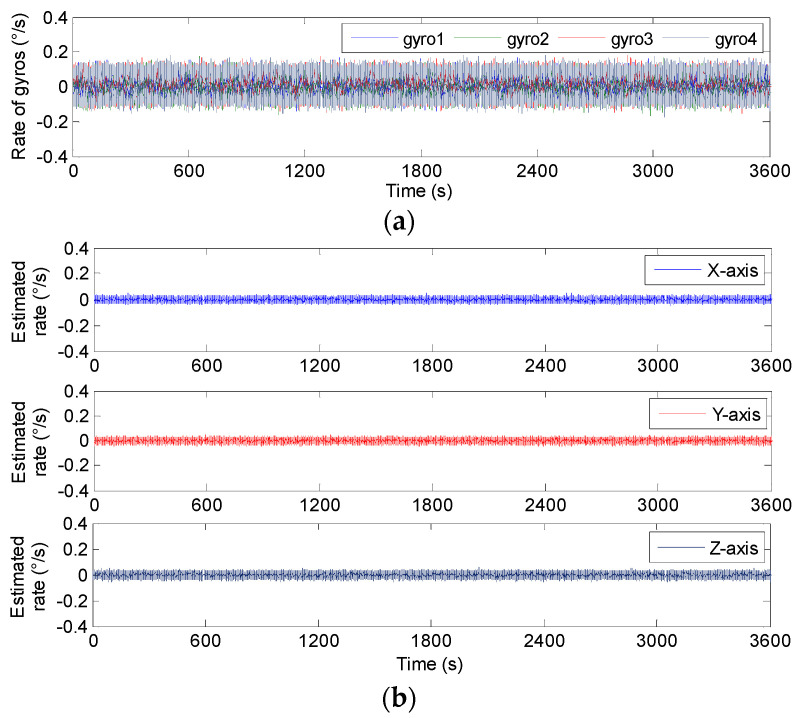
The static estimated results of 4-MIMU. (**a**) The outputs of gyros. (**b**) The estimated rate of the MIMU on the *X_b_*, *Y_b_* and *Z_b_* axes.

**Figure 22:**
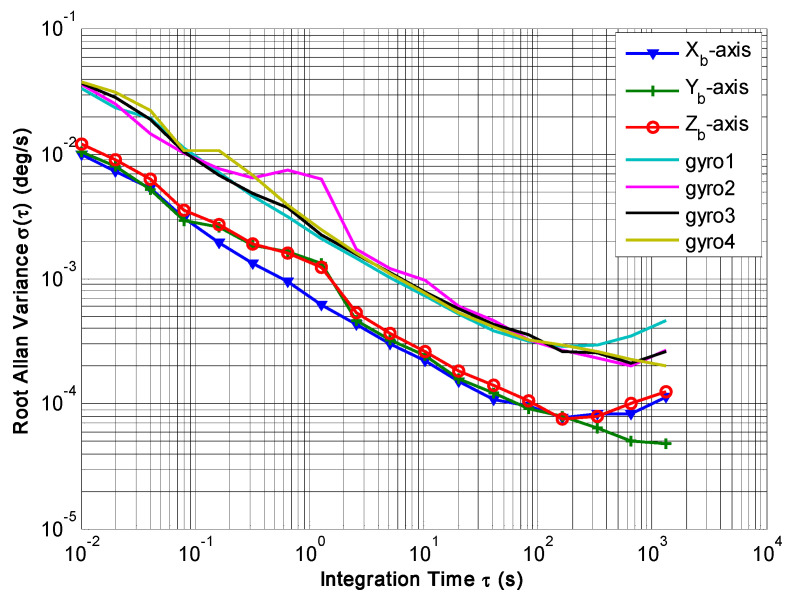
Plot of Allan variance between the gyros and fused signal rate in MIMU’s body coordinate frame.

The outputs of the 4-MIMU system were collected under static conditions, where the sampling time and period were set to 1 h and 0.01 s, respectively. Using the presented KF algorithm of Equation (9), the compared plot of signal rate and Allan variance are shown in Figure 21 and Figure 22, respectively. The results are listed in Table 8.

Figure 21 indicates that the estimated rate in MIMU’s body frame can be accurately obtained by fusing the outputs of four gyros in a redundant MIMU. In particular, Figure 22 shows that the Allan variance curves for a fused signal rate on the MIMU’s axes of *X_b_*, *Y_b_* and *Z_b_* are lower than that of a single gyroscope. In addition, Table 8 shows that the ARW and RRW for the fused signal rate are about 3.5 and 2.5 times lower than for single gyroscopes. The results demonstrate that the accuracy of the MIMU can be effectively improved by fusing of a non-orthogonal gyroscope array.

### 5.2. Swing Signal Testing Results

**Figure 23:**
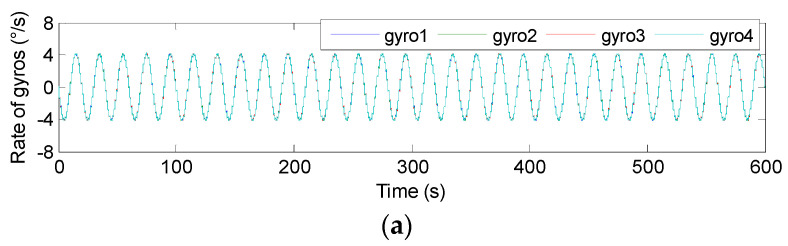

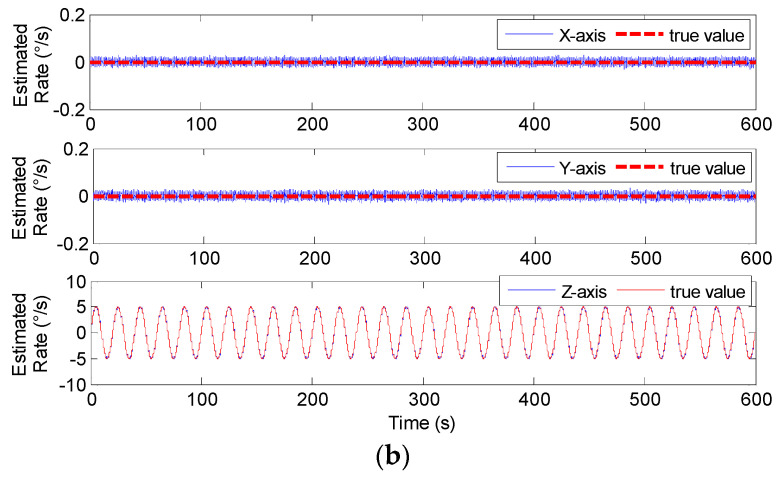
The swing test results of 4-MIMU with *ω_z_* = 5 × sin(2π*ft*)°*/s* (*f* = 0.05 Hz). (**a**) The outputs of gyros. (**b**) The estimated rate of the MIMU on the *X_b_*, *Y_b_* and *Z_b_* axes.

**Figure 24:**
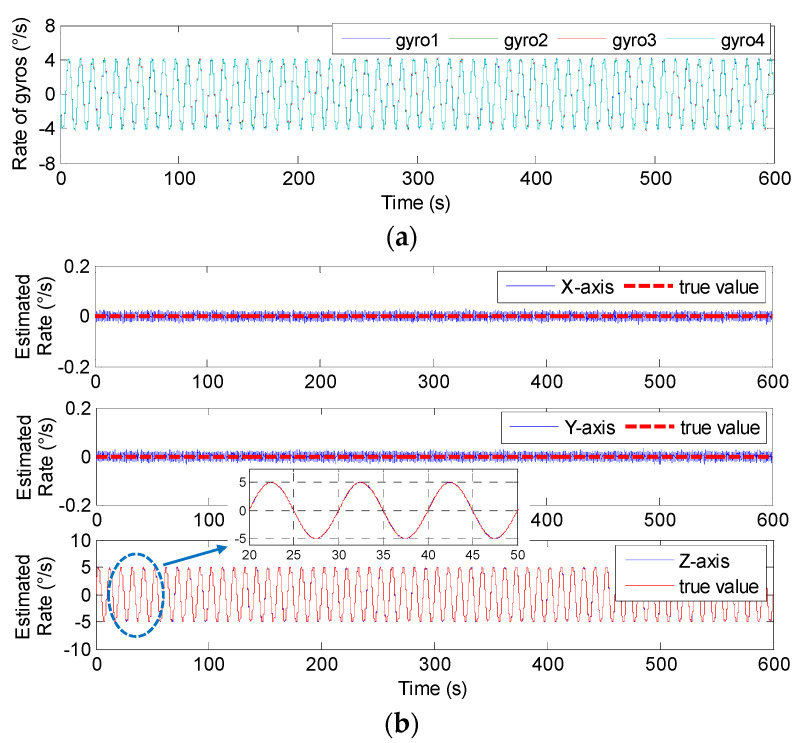
The swing test results of 4-MIMU with *ω_z_* = 5 × sin(2π*ft*)°*/s* (*f* = 0.1 Hz). (**a**) The outputs of gyros. (**b**) The estimated rate of the MIMU on the *X_b_*, *Y_b_* and *Z_b_* axes.

The swing test was carried out on a turntable, and the input signal rate was set to **ω***^b^*= [0, 0, 5 × sin(2π*ft*)]*^T^*°*/s* with *f* = 0.05 and 0.1 Hz. Therefore, the outputs of component gyroscopes and fused signal rate in MIMU’s body frame are shown in Figure 23 and Figure 24, and the estimated error is given in Table 9.

In Figure 23 and Figure 24, it can be seen that the amplitude of the signal rate on the MIMU’s *Z_b_* axis is similar to 5°*/s*, and Table 9 shows that the errors (1σ) on the MIMU’s *X_b_* and *Y_b_* axes were about 4.9 and 4.6 times lower than those of the single gyroscope. On the *Z_b_* axis, it was about 2.9-times lower. This demonstrates that the gyro’s error can be effectively reduced through fusing the outputs of the non-orthogonal array, thereby improving the accuracy of the MIMU and navigation system.

## 6. Conclusions

In this work, a redundant MIMU system was designed by a non-orthogonal gyro array, and an optimal fused KF algorithm was established by a steady-state gain to fuse array signals to improve the MIMU’s accuracy. In particular, two different conical configuration structures of a non-orthogonal array for 4, 5, 6 and 8 gyros were designed and analyzed. The results showed that *N*, the conical installation angle (*α*) and the correlation factor (*ρ*) will seriously affect the optimal configuration structures, eventually affecting the performance of the redundant MIMU system. The results also demonstrate that the input signal rate in the MIMU’s body frame could be effectively estimated, and the gyro’s error can be reduced. The experimental results of 4-MIMU illustrate that the gyro’s ARW and RRW can be decreased by factors of about 3.5 and 2.5 compared to the single gyro, respectively; and the estimated errors (1σ) on the MIMU’s *X_b_*, *Y_b_* and *Z_b_* axes were 4.9, 4.6 and 2.9 times lower than those of a single gyro in the swing test.

## Figures and Tables

**Table 1:** The GDOP of different configurations for non-orthogonal gyro array (*ρ* = 0).

Scheme	Angle *α*	*N* = 4	*N* = 5	*N* = 6	*N* = 8
GDOP (Scheme 1)	*α* = 60°	1.5327	1.3663	1.3089	1.0801
GDOP (Scheme 2)	*α* = 60°	1.5275	1.3540	1.2293	1.0609
GDOP (Scheme 2)	*α* = 45°	1.7512	1.5275	1.3732	1.1684

**Table 2:** The optimal angle *α* with different *N* and *ρ*.

Number	Correlation Factor	Minimum GDOP	Optimal Angle α
	*ρ* = 0.2	1.5269	49.94°
*N* = 4	*ρ* = −0.1	1.4671	57.72°
	*ρ* = −0.2	1.4117	61.75°
	*ρ* = 0.2	1.4000	49.10°
*N* = 5	*ρ* = −0.1	1.2845	58.71°
	*ρ* = −0.2	1.1798	65.68°
	*ρ* = 0.2	1.3076	48.36°
*N* = 6	*ρ* = −0.1	1.1450	59.85°
	*ρ* = −0.18	1.0160	69.11°
	*ρ* = 0.2	1.1802	47.05°
*N* = 8	*ρ* = −0.1	0.9353	62.93°
	*ρ* = −0.14	0.8050	75.57°

**Table 3:** Results of Allan variance of 6-gyro MIMU for Scheme 1.

Correlation Factor	MIMU Axis	ARW (°$/\sqrt{h}$)	RRW (°$/h/\sqrt{h}$)	BS (°/h)
*ρ* = −0.19	*X_b_*	0.0393	279.894	3.8459
	*Y_b_*	0.0396	256.254	3.8810
	*Z_b_*	0.0203	105.408	3.0038
*ρ* = 0	*X_b_*	0.0420	286.842	3.8645
	*Y_b_*	0.0414	281.496	3.8893
	*Z_b_*	0.0416	265.800	3.9016
*ρ* = 0.5	*X_b_*	0.0484	325.752	4.0414
	*Y_b_*	0.0480	313.020	3.9326
	*Z_b_*	0.0668	312.582	2.9394

**Table 4:** Results of Allan variance of 6-gyro MIMU for Scheme 2.

Correlation Factor	MIMU Axis	ARW (°$/\sqrt{h}$)	RRW (°$/h/\sqrt{h}$)	BS (°/h)
*ρ* = −0.19	*X_b_*	0.0398	243.432	3.8092
	*Y_b_*	0.0398	307.740	3.8684
	*Z_b_*	0.0675	95.526	2.9332
*ρ* = 0	*X_b_*	0.0412	292.770	3.9389
	*Y_b_*	0.0414	289.590	3.8406
	*Z_b_*	0.0418	266.388	3.9633
*ρ* = 0.5	*X_b_*	0.0443	255.594	3.9182
	*Y_b_*	0.0494	258.210	3.8985
	*Z_b_*	0.0476	302.604	4.3017

**Table 5:** The estimated errors of 6- and 8-gyro MIMU systems for Scheme 1.

Terms	Number	*X_b_*	*Y_b_*	*Z_b_*	Single Gyro
Estimated error (1*σ*, °/s)	*N* = 6	0.0142	0.0088	0.0224	0.0622
	*N* = 8	0.0111	0.0068	0.0203	0.0622
Reduction factor	*N* = 6	4.3803	7.0682	2.7768	
	*N* = 8	5.6036	9.1471	3.0640	

**Table 6:** The estimated error of 6- and 8-gyro MIMU systems for Scheme 2.

Terms	Number	*X_b_*	*Y_b_*	*Z_b_*	Single Gyro
Estimated error (1*σ*, °/s)	*N* = 6	0.0182	0.0107	0.0242	0.0552
	*N* = 8	0.0075	0.0075	0.0200	0.0552
Reduction factor	*N* = 6	3.0329	5.1589	2.2810	
	*N* = 8	7.3600	7.3600	2.7600	

**Table 7:** Cross-correlation matrix for a redundant 4-MIMU system.

Gyro Number	g1	g2	g3	g4
**g1**	1	0.000309	0.001531	−0.003095
**g2**	0.000309	1	0.001693	−0.001061
**g3**	0.001531	0.001693	1	−0.000106
**g4**	−0.003095	−0.001061	−0.000106	1

**Table 8:** Comparison results of the Allan variance measurement for 4-MIMU.

Gyro Number	$\mathrm{ARW}\;(\text{°}/\sqrt{h})$	$\mathrm{RRW}\;(\text{°}/h/\sqrt{h})$	BS (°/h)
gyro1	0.1476	4.979	1.0285
gyro2	0.1849	2.8545	0.9711
gyro3	0.1618	2.8764	0.9399
gyro4	0.1519	2.6229	0.9461
*X_b_*-axis	0.0429	1.1954	0.2751
*Y_b_*-axis	0.0459	0.6041	0.2287
*Z_b_*-axis	0.0525	1.3726	0.2723

**Table 9:** The estimated errors (1σ) of 4-MIMU for the swing test (*ω_z_* = 5 × sin(2π*ft*)).

Terms	Frequency *f*	*X_b_*	*Y_b_*	*Z_b_*
Estimated error (1*σ*, °/s)	*f* = 0.05	0.0074	0.0078	0.0125
	*f* = 0.10	0.0074	0.0079	0.0124
	*Single gyro*	0.0367	0.0367	0.0367
Reduction factor	*f* = 0.05	4.9595	4.7051	2.9360
	*f* = 0.10	4.9595	4.6456	2.9597

## Data Availability

Not applicable.
